# The cohesin acetylation cycle controls chromatin loop length through a PDS5A brake mechanism

**DOI:** 10.1038/s41594-022-00773-z

**Published:** 2022-06-16

**Authors:** Marjon S. van Ruiten, Démi van Gent, Ángela Sedeño Cacciatore, Astrid Fauster, Laureen Willems, Maarten L. Hekkelman, Liesbeth Hoekman, Maarten Altelaar, Judith H. I. Haarhuis, Thijn R. Brummelkamp, Elzo de Wit, Benjamin D. Rowland

**Affiliations:** 1 Division of Cell Biology, The Netherlands Cancer Institute, Amsterdam, the Netherlands; 2 Division of Biochemistry, Oncode Institute, The Netherlands Cancer Institute, Amsterdam, the Netherlands; 3 Proteomics Facility, The Netherlands Cancer Institute, Amsterdam, the Netherlands; 4 Biomolecular Mass Spectrometry and Proteomics, Bijvoet Center for Biomolecular Research, Utrecht Institute for Pharmaceutical Sciences, Utrecht University and Netherlands Proteomics Centre, Utrecht, the Netherlands; 5 Division of Gene Regulation, Oncode Institute, The Netherlands Cancer Institute, Amsterdam, the Netherlands

**Keywords:** Chromosomes, Chromatin structure

## Abstract

Cohesin structures the genome through the formation of chromatin loops and by holding together the sister chromatids. The acetylation of cohesin’s SMC3 subunit is a dynamic process that involves the acetyltransferase ESCO1 and deacetylase HDAC8. Here we show that this cohesin acetylation cycle controls the three-dimensional genome in human cells. ESCO1 restricts the length of chromatin loops, and of architectural stripes emanating from CTCF sites. HDAC8 conversely promotes the extension of such loops and stripes. This role in controlling loop length turns out to be distinct from the canonical role of cohesin acetylation that protects against WAPL-mediated DNA release. We reveal that acetylation controls the interaction of cohesin with PDS5A to restrict chromatin loop length. Our data support a model in which this PDS5A-bound state acts as a brake that enables the pausing and restart of loop enlargement. The cohesin acetylation cycle hereby provides punctuation in the process of genome folding.

## Abstract (web summary)

Here, the authors use gene editing combined with Hi-C to show that cohesin acetylation restricts the length of chromatin loops through enhanced binding of cohesin to PDS5A.

## Main

Cohesin plays an important role in three-dimensional (3D) genome organization through the formation and enlargement of chromatin loops^1–7^. This process requires the activity of cohesin’s ATPase and its regulator SCC2^NIPBL^ (refs. ^1–3,7^). Loop length is restricted by the cohesin release factor WAPL^3–5^. Together these proteins keep the looping process dynamic due to continuous cycles of cohesin-dependent formation of loops, their enlargement, and DNA release.

In mammals, the position of cohesin-dependent loops is determined by the architectural protein CTCF, which restricts chromatin loops to distinct chromosome domains, also known as topologically associated domains (TADs)^8–10^. Recent work shows that CTCF acts as an anchor point to stabilize cohesin on chromatin and promote the formation and/or maintenance of CTCF-anchored loops^11,12^. A CTCF mutant deficient in anchoring still displays TAD boundaries^11^, suggesting that anchoring may not fully explain the mechanism by which CTCF controls chromatin looping. CTCF appears also to act as a boundary to prevent passage beyond CTCF sites^11–14^, but whether this boundary function is mediated via cohesin-CTCF anchoring or a different molecular mechanism remains poorly understood.

The cohesin acetyltransferase ESCO1 acetylates the cohesin SMC3 subunit and localizes to CTCF sites^12,15,16^. ESCO1 was recently shown to stabilize cohesin on chromatin and promote the formation of CTCF-anchored loops. ESCO1 was proposed to do so by protecting cohesin against WAPL-mediated release^12^, similar to the mechanism by which cohesin is protected to maintain sister chromatid cohesion^9,17^. A study in budding yeast, however, showed that cohesin acetylation restricts chromatin loop length independently of WAPL^18^. The mechanism by which cohesin acetylation regulates chromatin looping therefore remains unclear.

While acetylation is important for locking cohesin on DNA, the role of deacetylation is less well understood. Cohesin deacetylation by the deacetylase HDAC8 (Hos1 in budding yeast) is required for recycling of cohesin complexes for the next round of sister chromatid cohesion^19–21^. HDAC8 is present throughout the cell cycle^21^, suggesting that it might play a role beyond recycling of cohesive cohesin complexes. If cohesin acetylation indeed stabilizes cohesin at CTCF sites, HDAC8-mediated deacetylation could provide a mechanism to enable further loop enlargement beyond CTCF.

In this study we explore the role of the cohesin acetylation cycle in controlling the 3D genome. We show that cohesin acetylation regulates chromosome folding by restricting the length of architectural stripes and chromatin loops. Cohesin acetylation appears to control genome organization independently of its canonical role in protecting against WAPL. We find that cohesin acetylation rather converts cohesin into a PDS5A-bound state to restrict the length of chromatin loops.

## Results

### Cohesin acetylation limits the length of stripes and loops

**Fig. 1:**
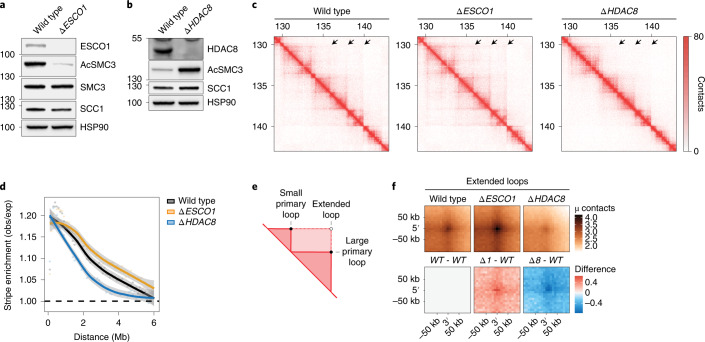
Cohesin acetylation restricts the length of architectural stripes and chromatin loops. **a**, Immunoblot analysis of *∆ESCO1* cells. The *∆ESCO1* cell line displays reduced levels of acetylated SMC3 (AcSMC3). This experiment was performed three times, with similar results. **b**, Immunoblot analysis of *∆HDAC8* cells; these cells have increased levels of acetylated SMC3. This experiment was performed three times, with similar results. **c**, Hi-C contact matrices for G1 cells of the indicated genotypes. A locus at chromosome 4 is shown at 20-kb resolution. Matrices were normalized to 100 million contacts per sample. The arrows indicate examples of architectural stripes whose length has changed in ∆*ESCO1* and ∆*HDAC8* cells. **d**, Aggregate stripe analysis to quantify signal enrichment emanating from CTCF sites at 100-kb resolution. Typically, interactions are formed close to the diagonal and decay over distance. These so-called expected contacts were obtained from a distance-normalized contact matrix; we then calculated enrichment of the observed (obs) contacts over the expected (exp) contacts at 100-kb resolution. This method reveals the presence of architectural stripes emanating from CTCF sites. *∆ESCO1* cells are enriched for long interactions while *∆HDAC8* cells display shorter interactions. **e**, Cartoon illustrating the difference between primary and extended loops. **f**, APA for extended loops. Differential APA plots for extended loops compared with wild type (WT). *∆ESCO1* (*∆1*) cells show an increase in extended loops while *∆HDAC8* (*∆8*) cells show a decrease. Source data

To assess whether the cohesin acetylation cycle regulates the 3D genome, we generated knockout cells for either ESCO1 or HDAC8 in the human HAP1 cell line using CRISPR–Cas9 technology (Fig. 1a,b). We found that ESCO1 in these cells is responsible for the vast majority of SMC3 acetylation (Fig. 1a) while *∆HDAC8* cells, as expected, exhibited increased levels of acetylated SMC3 (Fig. 1b). To specifically study the role of the cohesin acetylation cycle in chromatin looping, in contrast to its role in sister chromatid cohesion, we performed Hi-C analysis in G1-sorted cells. This also enabled us to assess looping in cells that lack cohesin acetylation, because cohesin’s other acetyltransferase, ESCO2, is absent in G1 (ref. ^22^).

**Extended Data Fig. 1:**
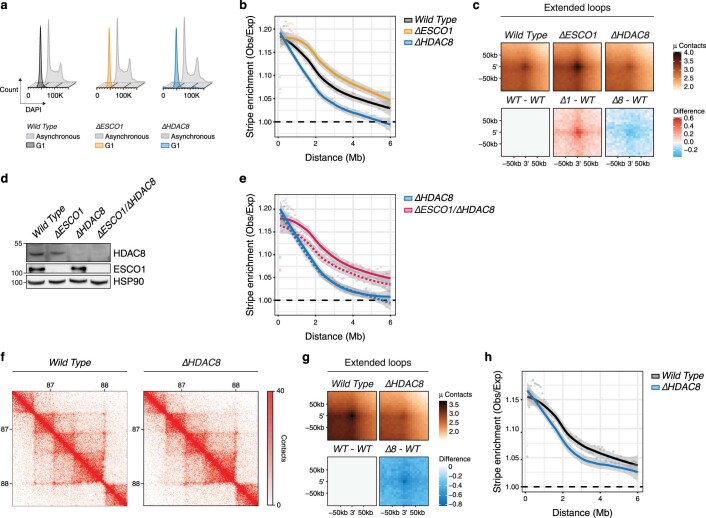
The looping defect of *∆HDAC8* cells is ESCO1-dependent. (**a**) The 10% smallest cells were sorted to obtain G1 cells. FACS plots showing the DNA content of unsorted (asynchronous) and sorted (G1) cells. Cells were fixed and stained with DAPI. (**b**) Aggregate stripe analysis to quantify the signal enrichment emanating from CTCF sites at 100-kb resolution. The architectural stripe phenotype is observed in independent clones. (**c**) APA analysis reveals that the extended loop phenotype is also observed in independent clones. Differential APA plots for extended loops compared to wild type (WT). *∆ESCO1* (*∆1*) cells show an increase in extended loops. *∆HDAC8* (*∆8*) cells show a decrease in extended loops. (**d**) Western blot analysis of the indicated genotypes. This experiment was performed twice with similar results. (**e**) Aggregate stripe analysis to quantify the signal enrichment emanating from CTCF sites at 100-kb resolution. The short stripes in *∆HDAC8* cells are rescued upon ESCO1 deletion. This phenotype is also observed in a replicate Hi-C experiment in an independent *∆ESCO1/∆HDAC8* clone (dashed line). (**f**) Hi-C contact matrices for asynchronous cells of the indicated genotypes. A locus at chromosome 2 is shown at 10-kb resolution. Matrices were normalized to 100 million contacts per sample. (**g**) APA for extended loops. Differential APA plots for extended loops compared to wild type (WT). Asynchronous *∆HDAC8* (∆8) cells show a decrease in extended loops. (**h**) Aggregate stripe analysis to quantify the signal enrichment emanating from CTCF sites at 100-kb resolution. Asynchronous *∆HDAC8* cells display shorter stripes in comparison to asynchronous wild type cells. Source data

Hi-C matrices of wild-type cells displayed expected features, including TADs, loops connecting CTCF sites, and architectural stripes. Such stripes are thought to be formed by monodirectional loop extrusion by cohesin that is anchored to CTCF. Interestingly, we observed notable differences in these stripes in cells with altered cohesin acetylation levels. While cells lacking ESCO1 showed an increase in the length of stripes, *∆HDAC8* cells displayed shorter stripes (Fig. 1c). To assess these stripes genome wide, we performed an aggregate stripe analysis that quantifies the distance of the contacts emanating from CTCF sites. These analyses showed that, relative to the wild type, the architectural stripes of *∆ESCO1* cells are enriched for longer-range contacts, while these are depleted in *∆HDAC8* cells (Fig. 1d and Extended Data Fig. 1b). We found that this looping defect in *∆HDAC8* cells is dependent on ESCO1 (Extended Data Fig. 1d,e). Cohesin acetylation thus seems to limit the length of architectural stripes.

We then investigated whether *∆ESCO1* cells harbor CTCF-anchored loops that extend even beyond those found in wild-type cells. We therefore scored the formation of ‘extended loops’, which are predicted to be formed when loops are enlarged beyond those computationally detected in wild-type cells (Fig. 1e)^3^. Extended loops indeed were more abundant in *∆ESCO1* cells (Fig. 1f), whereas such extended loops were decreased in *∆HDAC8* cells (Fig. 1f and Extended Data Figs. 1c and 5a,d). We note that the effects on architectural stripes were clearer than those on CTCF-anchored loops. The combined findings that *∆ESCO1* cells displayed longer architectural stripes and more pronounced extended loops, while *∆HDAC8* cells displayed shorter architectural stripes and loops, indicate that cohesin acetylation limits the degree to which loops can be enlarged.

### Acetylation controls loop length independently of WAPL

**Fig. 2:**
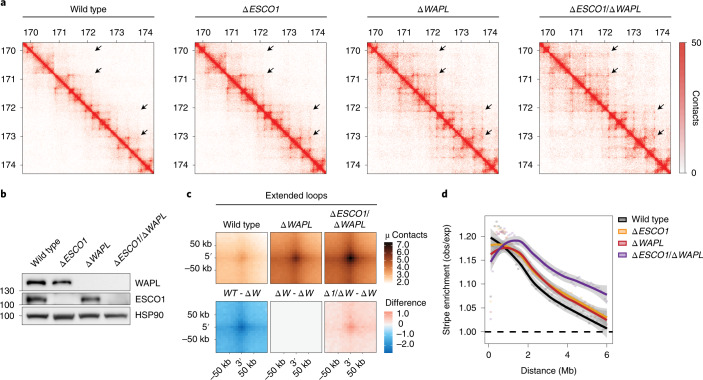
Cohesin acetylation restricts loop length in a WAPL-independent manner. **a**, Hi-C contact matrices for G1 cells of the indicated genotypes. A locus at chromosome 5 is shown at 10-kb resolution. Matrices were normalized to 100 million contacts per sample. **b**, Immunoblot analysis of the indicated genotypes. This experiment was performed twice, with similar results. **c**, APA for extended loops in the indicated genotypes (top); differential APA plots for extended loops compared with *∆WAPL* (*∆W*) cells (bottom). *∆ESCO1*/*∆WAPL* (*∆1*/*∆W*) cells show an increase in extended loops in comparison with *∆WAPL* cells. **d**, Aggregate stripe analysis to quantify signal enrichment emanating from CTCF sites at 100-kb resolution. Both *∆ESCO1* and *∆WAPL* cells display longer stripes in comparison with wild-type cells, although stripes are extended even further in *∆ESCO1*/*∆WAPL* cells. Source data

**Extended Data Fig. 2:**
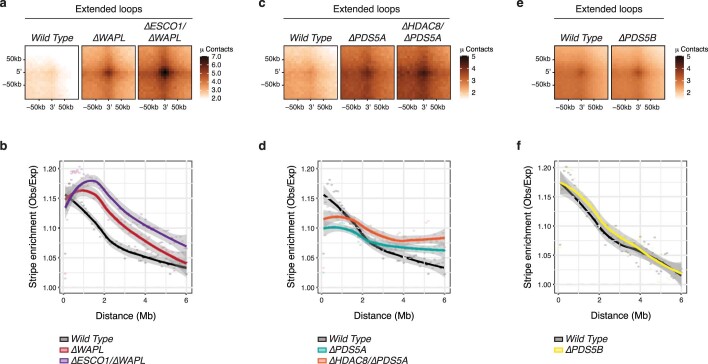
Hi-C replicates in G1 cells. (**a**) ﻿Aggregate peak analysis (APA) reveals that the extended loop phenotype is also observed in a replicate Hi-C experiment. *∆ESCO1/∆WAPL* cells show an increase in extended loops in comparison to *∆WAPL* cells. (**b**) The extended stripe phenotypes for *∆WAPL* and *∆ESCO1/∆WAPL* cells are also observed in a replicate Hi-C experiment. The aggregate stripe analysis quantifies the signal enrichment emanating from CTCF sites at 100-kb resolution. (**c**) APA plots show that the extended loop phenotypes are also observed in a replicate Hi-C experiment. For *∆PDS5A* (*∆5* *A*) cells we used an independent clone, for *∆HDAC8/∆PDS5A* (*∆8/∆5A*) cells we used the same clone. (**d**) The extended stripe phenotypes for *∆PDS5A* and *∆HDAC8/∆PDS5A* cells are also observed in a replicate Hi-C experiment in the clones described in (c). The aggregate stripe analysis quantifies the signal enrichment emanating from CTCF sites at 100-kb resolution. (**e**) APA in an independent *∆PDS5B* clone confirms that PDS5B does not regulate the formation of extended loops. (**f**) Hi-C analysis in an independent *∆PDS5B* clone confirms that PDS5B does not regulate the length of architectural stripes.

Previous studies revealed that the cohesin-release factor WAPL restricts the extension of chromatin loops^3,4^. We show that ESCO1, to some degree, is also important in restricting the size of chromatin loops. If cohesin acetylation simply protects cohesin against WAPL-mediated release, one would not expect to find an increase in long-range interactions in *∆ESCO1* cells. To directly test whether ESCO1 and WAPL might regulate looping independently, we generated double-knockout cells for *ESCO1* and *WAPL*, and performed Hi-C analyses in G1-sorted cells (Fig. 2a,b). These analyses revealed that deletion of *ESCO1* exacerbates the *∆WAPL* phenotype (Fig. 2a). In comparison with *∆WAPL* cells, *∆ESCO1/∆WAPL* cells displayed longer architectural stripes and harbored more pronounced extended loops (Fig. 2c,d and Extended Data Fig. 2a,b). We thus find that cohesin acetylation restricts the size of chromatin loops in a manner that is at least partially independent of WAPL.

### Acetylation converts cohesin into a PDS5A-bound state

**Fig. 3:**
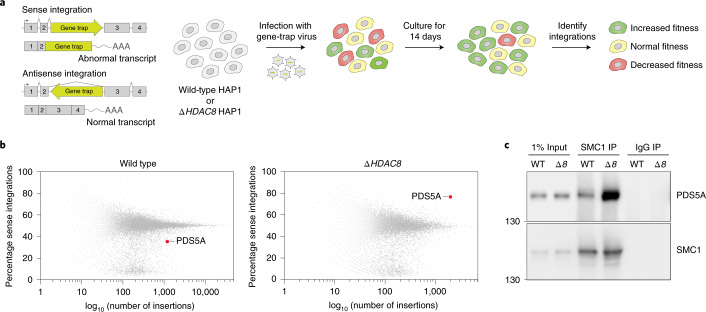
Cohesin acetylation converts cohesin into a PDS5A-bound state. **a**, Schematic overview of the setup of the haploid genetic screen, comparing control HAP1 cells with *∆HDAC8* HAP1 cells. This genome-wide genetic screen involves the infection of haploid HAP1 cells with a gene-trap virus, leading to a polyclonal collection of knockout cells. Intronic insertion of the gene trap in a sense orientation creates a knockout of the affected gene, while intronic insertion in the antisense orientation does not affect it. If loss of a gene is beneficial for cellular outgrowth, sense insertions will be enriched over time. **b**, Plot depicting screen results for wild-type and *∆HDAC8* cells. Each dot represents one gene. The percentage of sense integrations in comparison to the total amount of insertions shows the importance of each gene for cell viability. Upward shift in the cloud indicates that loss of PDS5A is beneficial specifically for *∆HDAC8* cells. **c**, Pulldown experiment on the core cohesin subunit SMC1, revealing an increase in PDS5A binding to cohesin in *∆HDAC8* (*∆8*) cells. This experiment was performed three times, with similar results. Source data

**Extended Data Fig. 3:**
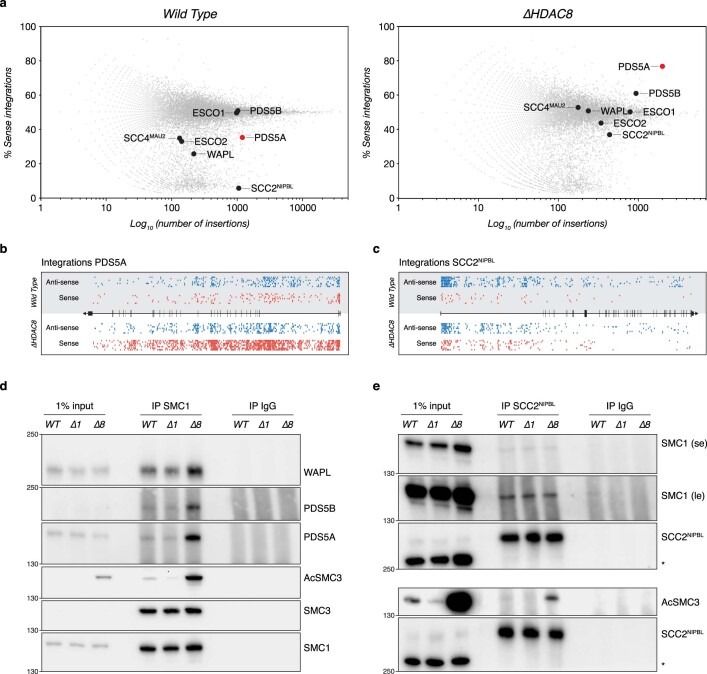
Haploid genetic screen in ∆*HDAC8* cells. (**a**) Plot depicting the screen results for wild type cells and *∆HDAC8* cells. Several cohesin regulators are highlighted. (**b**) Gene-trap integration patterns for PDS5A in anti-sense (blue) or sense (red) orientation in wild type and *∆HDAC8* cells. *∆HDAC8* cells harbour an increase in sense insertions along the entire gene. (**c**) Gene-trap integration patterns for SCC2^NIPBL^ in anti-sense (blue) or sense (red) orientation in wild type and *∆HDAC8* cells. The sense insertions in *∆HDAC8* cells appear to be tolerated until exon 10, while exons 11 - 47 appear to remain essential. This pattern much resembles the pattern found in *∆WAPL* cells^3^. (**d**) Pulldown experiment on the core cohesin subunit SMC1 in cells lacking *ESCO1* (*∆1*) or *HDAC8* (*∆8*). We find that cohesin in *∆HDAC8* cells is enriched for binding to PDS5A, PDS5B, and WAPL. Cohesin’s binding to these factors appears to be less evidently affected in *∆ESCO1* cells. We note that in wild type cells only a small fraction of cohesin complexes is acetylated. These low acetylation levels could explain why it is relatively difficult to assess differences in binding of the mentioned proteins in *∆ESCO1* cells. This experiment was performed 3 times with similar results. (**e**) Pulldown experiment on the cohesin regulator SCC2^NIPBL^ in cells lacking *ESCO1* (*∆1*) or *HDAC8* (*∆8*). The upper three rows belong to one experiment and the lower two rows to another experiment. Both a short exposure (se) and long exposure (le) are shown for the core cohesin subunit SMC1. We find that the amount of cohesin acetylation does not affect SCC2^NIPBL^ binding to cohesin. We also find that SCC2^NIPBL^ pulls along acetylated cohesin complexes, suggesting that cohesin acetylation and cohesin’s binding to SCC2^NIPBL^ are not mutually exclusive. This experiment was performed 3 times with similar results. Source data

To identify which factors are key to the mechanism by which cohesin acetylation controls loop length, we performed a haploid genetic screen^23^. To find genetic interactors of HDAC8, we compared control HAP1 cells with *∆HDAC8* HAP1 cells (Fig. 3a). This screen revealed that *∆HDAC8* cells specifically benefit from losing PDS5A (Fig. 3b, Extended Data Fig. 3a,b and Supplementary Table 3). PDS5A is a regulatory cohesin subunit that inhibits cohesin’s ATPase activity^24,25^. To test whether cohesin acetylation affects the binding of cohesin to PDS5A, we performed coimmunoprecipitation experiments. Pulldown of the core cohesin component SMC1 revealed that PDS5A is more frequently bound to cohesin in *∆HDAC8* cells in comparison with wild-type cells (Fig. 3c and Extended Data Fig. 3d). Cohesin acetylation apparently converts cohesin into a PDS5A-bound state. It remains unclear whether we identified PDS5A as a hit in our screen due to a role for this factor in DNA looping, or rather due to a role in for example sister chromatid cohesion.

### Cohesin acetylation controls loop length through PDS5A

**Fig. 4:**
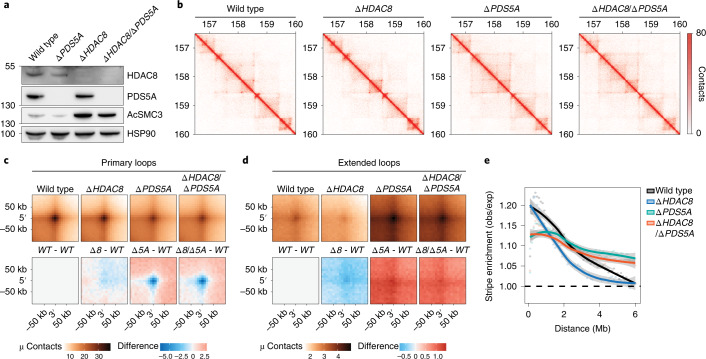
Cohesin acetylation controls loop length through PDS5A. **a**, Immunoblot analysis of the indicated genotypes. This experiment was performed three times, with similar results. **b**, Hi-C contact matrices for G1 cells of the indicated genotypes. A locus at chromosome 5 is shown at 10-kb resolution. Matrices were normalized to 100 million contacts per sample. **c**, APA for primary loops (top). The bottom row displays differential APA plots for primary loops in *∆HDAC8* (*∆8*) cells, *∆PDS5A* (*∆5* *A*) cells and *∆HDAC8/∆PDS5A* (*∆8*/*∆5A*) cells in comparison with wild-type (WT) cells. *∆PDS5A* and *∆HDAC8*/*∆PDS5A* cells show a decrease in primary loops. **d**, APA for extended loops (top). The bottom row displays differential APA plots for extended loops compared with wild type. *∆HDAC8* cells show a decrease in extended loops; this defect was rescued following deletion of PDS5A. *∆PDS5A* and *∆HDAC8*/*∆PDS5A* cells both show an increase in extended loops. **e**, Aggregate stripe analysis to quantify signal enrichment emanating from CTCF sites at 100-kb resolution. Deletion of PDS5A in *∆HDAC8* cells rescued the shorter interactions found in *∆HDAC8* cells. Source data

To assess whether PDS5A is key to the mechanism by which cohesin acetylation restricts loop length, we deleted *PDS5A* in *∆HDAC8* cells (Fig. 4a). Hi-C analyses on G1-sorted cells revealed that *∆HDAC8*/*∆PDS5A* cells display an increase in both extended loops and the length of architectural stripes, which is comparable to what is observed in *∆PDS5A* cells (Fig. 4b–e and Extended Data Fig. 2c,d). Notably, *∆HDAC8*/*∆PDS5A* cells retained high levels of cohesin acetylation (Fig. 4a). Together, these findings suggest that cohesin acetylation by itself does not prevent loop enlargement, and that the restrictive role of cohesin acetylation in DNA looping requires PDS5A.

Interestingly, single knockouts for *PDS5A* already displayed a distinct chromatin looping phenotype (Fig. 4b). *∆PDS5A* cells showed an increase in extended loops, which appeared to be at the expense of primary loops (Fig. 4c,d). The difference plot, however, revealed that this increase in long-range interactions is not specific to CTCF sites, as we did not see a clear focal enrichment at the loop anchor (Fig. 4d). Likewise, *∆PDS5A* cells showed an increase in the length of stripes, while this signal was less enriched close to CTCF sites (Fig. 4e). These findings suggest that PDS5A not only promotes the formation of CTCF-anchored loops, but also restricts the enlargement of chromatin loops genome wide.

### No prominent role for PDS5B in 3D genome organization

**Extended Data Fig. 4:**
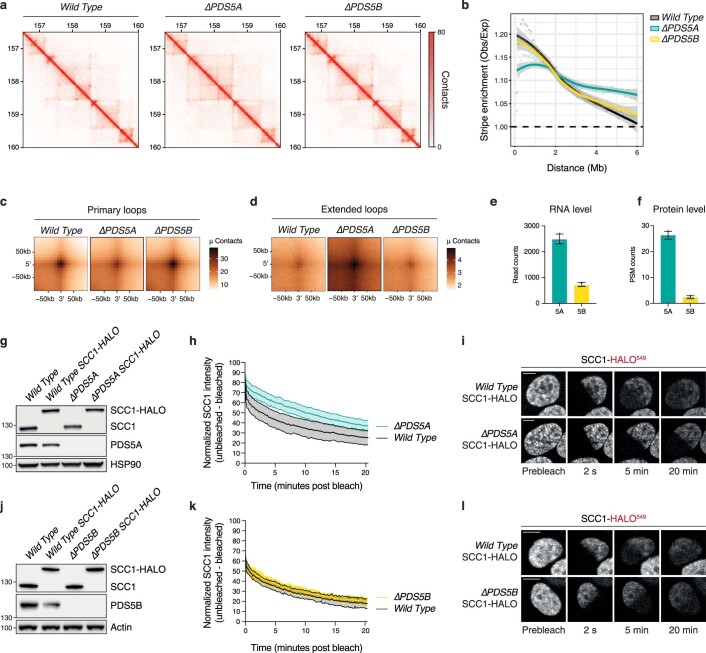
PDS5B does not control chromatin looping in HAP1 cells. (**a**) Hi-C contact matrices for G1 cells of the indicated genotypes. A locus at chromosome 5 is shown at 10-kb resolution. Matrices were normalized to 100 million contacts per sample. *∆PDS5B* cells do not display chromatin looping defects. (**b**) Aggregate stripe analysis to quantify the signal enrichment emanating from CTCF sites at 100-kb resolution. PDS5B does not control the length of stripes. (**c**) Aggregate peak analysis (APA) for primary loops. PDS5B does not regulate primary loops. (**d**) APA for extended loops. *∆PDS5B* cells do not show an increase in extended loops. (**e**) The RNA read counts of PDS5A and PDS5B in wild type HAP1 cells, from^11^. Mean and standard deviation are shown of three biological replicates (grey circles depict replicates). (**f**) The PSM counts of PDS5A and PDS5B in whole cell proteomics in wild type HAP1 cells. Mean and standard deviation are shown of three biological replicates (grey circles depict replicates). (**g**) Western blot analysis of wild type and *∆PDS5A* cells with either untagged or tagged SCC1-HALO. (**h**) Quantification of the FRAP experiment in SCC1-HALO tagged G1 cells. Mean and standard deviation for 17 wild type cells and 17 *∆PDS5A* cells, measured over 5 independent experiments. (**i**) Example images of cells used in (h) at the indicated time points after photobleaching. White scale bar is 5 µm. Note that the ∆*PDS5A* cells display a ‘vermicelli’-like SCC1 localization. (**j**) Western blot analysis of wild type and *∆PDS5B* cells with either untagged or tagged SCC1-HALO. (**k**) Quantification of the FRAP experiment in SCC1-HALO tagged G1 cells. Mean and standard deviation for 12 wild type cells and 12 *∆PDS5B* cells, measured over 4 independent experiments. (**l**) Example images of cells used in (k) at the indicated time points after photobleaching. White scale bar is 5 µm. Source data

**Extended Data Fig. 5:**
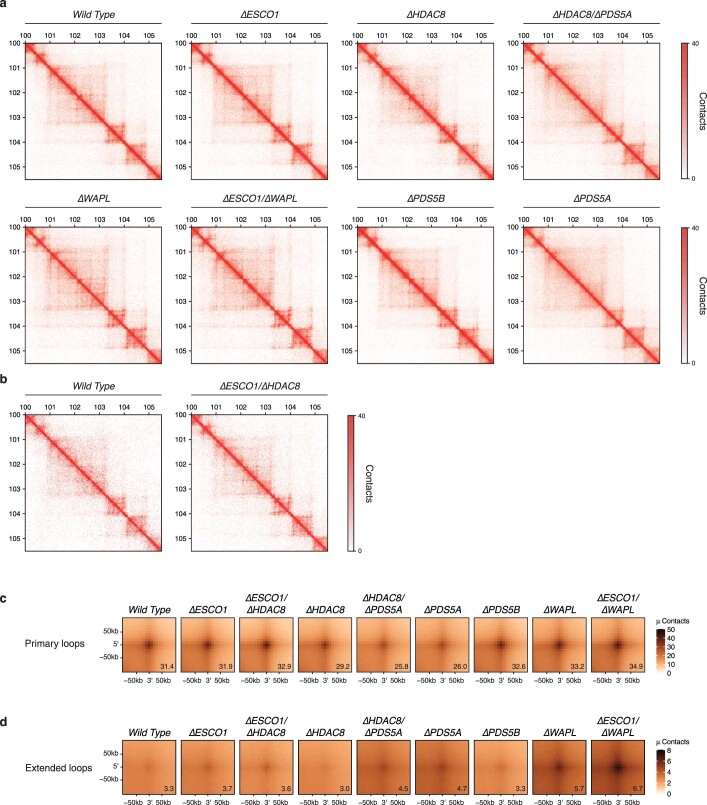
Overview Hi-C analyses of different genotypes. (**a**) Hi-C contact matrices for G1 cells of the indicated genotypes. A locus at chromosome 4 is shown at 10-kb resolution. Matrices were normalized to 100 million contacts per sample. (**b**) Hi-C contact matrices for G1 cells of the indicated genotypes. A locus at chromosome 4 is shown at 10-kb resolution. Matrices were normalized to 100 million contacts per sample. These Hi-C libraries were less deeply sequenced than the Hi-C libraries presented in (a). (**c**) Aggregate peak analysis (APA) for primary loops using the same scale for all genotypes. The bottom right value depicts the APA score. (**d**) APA for extended loops using the same scale for all genotypes. The bottom right value depicts the APA score.

Cells deficient for the PDS5A paralog PDS5B displayed no evident changes in primary loops, extended loops, or the length of architectural stripes (Extended Data Fig. 4a–d and Extended Data Fig. 2e,f). Our observation that only *∆PDS5A* cells, and not *∆PDS5B* cells, display such phenotypes could be explained by differences in abundance, because we found that PDS5A is considerably more abundant than PDS5B (Extended Data Fig. 4e,f). Chromatin looping in HAP1 cells thus appears to be largely controlled by PDS5A.

## Discussion

**Fig. 5:**
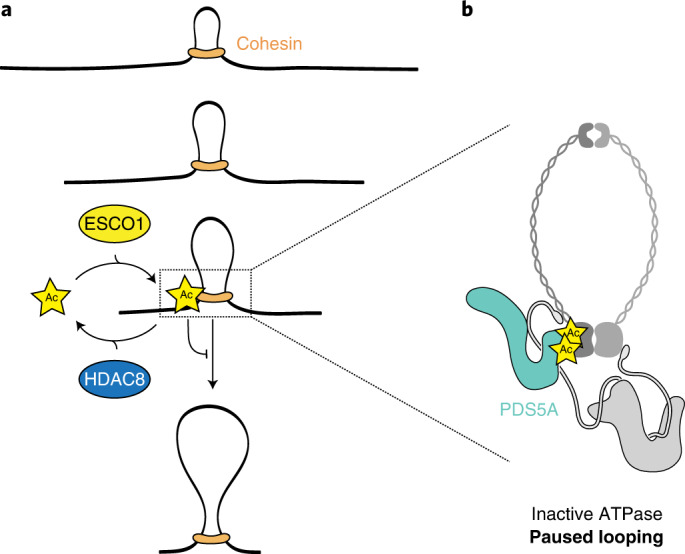
Model of how the cohesin acetylation cycle controls chromatin loop length. **a**, Cohesin acetylation regulates the size of architectural stripes and loops. Our data support a model in which ESCO1 acetylates SMC3 to pause cohesin looping while HDAC8 deacetylates cohesin to promote further loop enlargement. **b**, Cohesin acetylation converts cohesin into a PDS5A-bound state. We propose that this PDS5A binding to cohesin could inactivate cohesin’s ATPase to pause the looping process. Deacetylation by HDAC8 could alleviate this brake to restart the looping reaction.

Taken together, we find that the cohesin acetylation cycle regulates genome folding, and does so by modulating the length of loops and architectural stripes. While ESCO1 prevents the extension of such loops and stripes, HDAC8 promotes this extension (Fig. 5a). This role in controlling loop length turns out to be distinct from the canonical role of cohesin acetylation that protects against WAPL-mediated DNA release. We show that the cohesin acetylation cycle instead controls the binding of PDS5A to regulate loop enlargement.

Our findings indicate that cohesin acetylation regulates the looping process at CTCF sites through PDS5A. These findings fit with earlier work in HeLa cells showing that combined depletion of PDS5A and PDS5B decreased the amount of CTCF loops^4^. Importantly, we find that cohesin acetylation and PDS5A do not control the presence of architectural stripes, but rather their length. This suggests that the acetylation cycle does not control CTCF anchoring by itself. It is more likely that a PDS5A-dependent brake mechanism allows for the pausing of loop enlargement at CTCF sites. This mechanism in turn could enable CTCF to act as a boundary, albeit transiently. Such a PDS5A brake mechanism appears to not be limited to CTCF sites, because Pds5 likewise inhibits the loop enlargement process in yeast, which have no CTCF^18,26^. We suggest that cohesin acetylation and PDS5 binding thus represent an ancient regulatory mechanism, which is taken advantage of by CTCF to control loop length.

But how, mechanistically speaking, could the cohesin acetylation cycle and PDS5A binding then control looping? Earlier work showed that SCC2^NIPBL^ stimulates cohesin’s ATPase, whereas PDS5 inhibits cohesin’s ATPase activity^24,25^. This fits well with the finding that cohesin can initiate and enlarge DNA loops only in the presence of SCC2^NIPBL^^1,2^ but not in the presence of PDS5 (ref. ^1^). Because PDS5 and SCC2^NIPBL^ compete for the same binding interface on SCC1 (refs. ^25,27^), regulation of this exchange could provide a mechanism to control the loop enlargement process.

Recent structural work reveals that SCC2^NIPBL^ binds cohesin at multiple interfaces, including SMC3’s ATPase head^28–32^. Binding to this latter interface is observed in cohesin’s unacetylated state^28–30^. A key part of this interface contains the two lysines that are acetylated by cohesin’s acetyltransferases. Acetylation of these lysines neutralizes their charge and has been proposed to disfavor interaction with SCC2^NIPBL^ (ref. ^30^). However, mutant cohesin complexes in which these lysines are replaced by ‘acetyl-mimicking’ glutamines are barely impaired in their ATPase activity^29^. We indeed found that SCC2^NIPBL^ can still bind to acetylated cohesin complexes (Extended Data Fig. 3e). Together this would indicate that cohesin acetylation does not intrinsically compromise SCC2^NIPBL^ binding, and that cohesin acetylation can be compatible with ATPase activity.

Correspondingly, we found that cohesin acetylation by itself does not restrict chromatin looping, but only does so in the presence of PDS5A. Cryo-EM structures of yeast cohesin show that Pds5 binds to the Smc3 ATPase head^32^. Our data suggest that acetylation of SMC3 actually enhances the binding of cohesin to PDS5A. Further support for this model comes from observations in yeast that nonacetylatable Smc3 mutants display reduced Pds5 binding (see the paper by Bastié et al.^33^), and that acetylation promotes the stability of Pds5 on chromatin^34,35^.

The interconnection between SCC2^NIPBL^ and PDS5, and how these proteins regulate the activities of cohesin, remains incompletely understood. Future studies will be needed to assess whether PDS5 binding to acetylated cohesin indeed prevents SCC2^NIPBL^ binding to the SMC3 ATPase head, and whether SCC2^NIPBL^ may then remain connected to the complex through another interface. It would also be relevant to test whether acetylation-dependent PDS5 binding indeed inhibits SCC2^NIPBL^-stimulated ATPase activity. Such experiments could include in vitro ATPase assays on acetylated cohesin complexes, in which the amount of PDS5 is titrated until SCC2^NIPBL^-stimulated ATPase activity is inhibited, similar to previous experiments using unacetylated cohesin complexes^32^.

While questions remain regarding SCC2^NIPBL^, our data support the model where the binding of PDS5 to cohesin stabilizes the complex in a conformation that prevents ATP hydrolysis and further loop enlargement. By promoting the binding of PDS5, cohesin acetylation could maintain this conformation and thereby convert enlarging loops into static loops. HDAC8-mediated deacetylation in turn could alleviate this paused state to restart the looping reaction (Fig. 5b). This key regulatory mechanism turns out to be conserved from yeast to humans (see the paper by Bastié et al.^33^). The modulation of cohesin’s looping activity by cohesin acetylation could therefore be a universal mechanism that controls genome topology across the eukaryotic tree of life.

## Methods

### Cell culture and gene editing

HAP1 cells^36^ were cultured in Iscove’s modified Dulbecco’s medium (Invitrogen) supplemented with 10% fetal calf serum (FCS; Clontech), 1% UltraGlutamin (Lonza) and 1% penicillin-streptomycin (Invitrogen). Knockout cell lines were generated either by insertion of a resistance cassette in the first coding exon after the start ATG or by generation of small out-of-frame indels using CRISPR–Cas9 technology. CRISPRs targeting *ESCO1* (5’-CATGAGTACAAGGTCATCAA-3’ and 5’-AGCTTAACCGGAGATCACAA-3’), *HDAC8* (5’-CAGTGGGCAGTCGCTGGTCC-3’ and 5’-CGGGACTATAGATATAAACC-3’), *PDS5A* (5’-GTGGCGTCGTGAGTGCCGACGGG-3’ and 5’-GGAAGATCGCTTACCCTCCG-3’) and *PDS5B* (5’-TCTGATATTTCCTTGACCCC-3’) were cloned into px330 (Addgene plasmid no. 42230). *∆WAPL* cells were generated as previously described^3^, and used as a parental cell line to generate double-knockout cells for *WAPL* and *ESCO1*. Either blasticidin or puromycin resistance cassettes were used, as described previously^23^. Knockout cell lines were confirmed by PCR genotyping and immunoblotting analysis. The following oligos were used for ESCO1: 5’-CCAGGACACAAAAATCCTCTTC-3’ and 5’-CTTCATCTCATTCTTTTTCGGG-3’; for HDAC8: 5’-TAGGGCAACAAGGATGGTTAGT-3’ and 5’-TTTCTTGGGATTACAGGCAGAT-3’; for PDS5A: 5’-ACTGTGAACCAAAAGTTGTCCC-3’ and 5’-ATCAAAATCCGTCCAGACACTT-3’; and for PDS5B: 5’-GTTACAAATTTTGGTTGGTGGG-3’ and 5’-CCTCTGCCCTACACAGATGTAA-3’. A list of cell lines used in this manuscript is given in Supplementary Table 1.

To endogenously tag the C terminus of SCC1^RAD21^ with a HALO-tag, we used an approach previously described^32^ with slight adaptations. Because *∆PDS5A* and *∆PDS5B* cells were already resistant to puromycin, we cotransfected pRS-BLAST at a 1:10 ratio to the px459 plasmid. Transfected clones were selected using 10 μg ml^–1^ blasticidin for 2 days. Colonies were picked when clearly visible. Integration of the HALO-tag at the correct location was then confirmed by PCR genotyping and immunoblotting analysis. Wild-type cells with SCC1-HALO tagged were generated as previously described^32^.

### Immunoblotting

Cells were pelleted at 500*g* for 3 min and resuspended in RIPA buffer consisting of 10 mM Tris-Cl (pH 8.0), 1 mM EDTA, 0.5 mM EGTA, 1% Triton X-100, 0.1% sodium deoxycholate, 0.1% SDS and 140 mM NaCl, supplemented with protease inhibitors (Roche). Lysates were vortexed for 30 s and incubated on ice for 30 min. Lysates were spun at 20,000*g* for 10 min at 4 °C, and supernatants were quantified by Bradford analysis (Bio-Rad). Denatured proteins (10–20 μg) were loaded and run on polyacrylamide gels and transferred to nitrocellulose membranes. Membranes were blocked with 5% w/v milk in TBS-Tween (TBS-T, 0.1%). Primary and secondary antibody incubations were performed in 5% milk in TBS-Tween (0.1%) and membranes were washed with TBS-T, except for SCC2^NIPBL^, which was incubated in 5% bovine serum albumin (Sigma) in TBS-T (0.1%). The signal was developed with Clarity Western ECL Substrate (Bio-Rad) or Immobilon western Chemiluminescent HRP substrate (Millipore) on the ChemiDoc Imaging System (Bio-Rad).

### Coimmunoprecipitation

Cells were pelleted at 500*g* for 5 min and resuspended in lysis buffer consisting of 50 mM Tris (pH 7.5), 5 mM EDTA, 150 mM NaCl and 0.1% NP-40, supplemented with protease inhibitors (Roche) and phosphatase inhibitors (Sigma, 1:100). Cells were incubated for 30 min on ice. Lysates were supplemented with Ambion DNase I (Invitrogen, 1:100) and Benzonase Nuclease (Millipore, 600 U ml^–1^) and incubated for 4 h on a rotator at 4 °C. Lysates were spun at 20,000*g* for 10 min at 4 °C and supernatants were mixed with two volumes of TNENG buffer, consisting of 50 mM Tris (pH 7.5), 5 mM EDTA, 150 mM NaCl, 0.1% NP-40 and 10% glycerol, supplemented with protease inhibitors (Roche). Supernatants were quantified by Bradford analysis (Bio-Rad). Protein lysate (300 μg for SMC1 IP and 750 μg for SCC2 IP) was used, along with 3 μg of antibody. Lysates were mixed with 30 μl of Protein G Dynabeads (Invitrogen) and incubated overnight at 4 °C while tumbling. Beads were washed three times with wash buffer consisting of 50 mM Tris (pH 7.5), 5 mM EDTA, 150 mM NaCl and 0.1% NP-40, and proteins were denatured using Laemmli buffer at 95 °C for 10 min. Coimmunoprecipitation was checked by immunoblotting analysis, as described above.

### Antibodies

Coimmunoprecipitation experiments were performed with the following antibodies: SMC1 (Bethyl, no. A300-055A) and SCC2 (Bethyl, no. A301-779A). Immunoblots were performed using the following antibodies and dilutions: HSP90 (Santa Cruz, no. sc13119 F8, 1:10,000), ESCO1 (a kind gift from S. Rankin^37^, 1:1,500), HDAC8 (Sigma-Aldrich, no. WH0055869M1, 1:1,000), AcSMC3 (a kind gift from K. Shirahige^38^, 1:1,500), WAPL (Santa Cruz, no. sc365189, 1:1,000), SMC1 (Bethyl, no. A300-055A, 1:2,000), SMC3 (Bethyl, no. A300-060A-5, 1:2,000), SCC1 (Millipore, no. 05-908, 1:1,000), PDS5A (Bethyl, no. A300-089A, 1:1,000), PDS5B (Bethyl, no. A300-538A, 1:500), SCC2 (Santa Cruz, no. sc374625, 1:1,000), SCC4 (Abcam, no. ab46906, 1:1,000), Actin (Abcam, no. ab6276, 1:5,000) and Tubulin (Abcam, no. ab18251, 1:10.000). Secondary antibodies Goat-anti-Mouse-PO (DAKO, no. P0447) and Goat-anti-Rabbit-PO (DAKO, no. P0448) were used at 1:2,000 dilution.

### Fluorescence recovery after photobleaching

Cells with endogenously tagged SCC1 were grown on LabTekII-chambered cover glass (Thermo Scientific Nunc). To be able to specifically perform fluorescence recovery after photobleaching (FRAP) on G1 cells, cells were transfected with DNA helicase B fragment fused with near-infrared fluorescent protein (DHB-iRFP) using FuGENE Transfection Reagent 2–3 days before imaging. On the day of imaging, cells were incubated for 30 min with 300 nM HALO-ligand JF549 (Promega). Cells were washed three times with normal medium and incubated for 30 min to allow removal of excess ligand. The medium was replaced with Leibovitz L-15 imaging medium (Invitrogen), then FRAP analysis was performed on a Leica SP5 confocal microscope with a ×63/1.4 numerical aperture oil objective using the LAS-AF FRAP-Wizard. G1 cells were selected based on nuclear localization of DHB-iRFP, as described in ref. ^11^. Five images were taken before bleaching, then half of the nucleus was photobleached using five pulses of 100% transmission of the 561-nm laser. After bleaching, ten frames were taken every 2 s and subsequently 120 frames were taken every 10 s. Fluorescence intensity was measured in bleached and unbleached areas by user-defined regions in ImageJ v.2.1.0/1.53k. Recovery was quantified by calculating the difference in intensity between bleached and unbleached regions. To ensure that we quantified cells with homogeneous SCC1 distribution, we excluded those in which the difference in intensity between bleached and unbleached areas was already >10% in prebleaching frames.

### MS analysis

For protein digestion, frozen cell pellets were lysed in boiling guanidine (GuHCl) lysis buffer as previously described^39^. Protein concentration was determined with a Pierce Coomassie (Bradford) Protein Assay Kit (Thermo Scientific), according to the manufacturer’s instructions. After dilution to 2 M GuHCl, aliquots corresponding to 200 μg of protein were digested twice (4 h and overnight) with trypsin (Sigma-Aldrich) at 37 °C, enzyme/substrate ratio 1:75. Digestion was quenched by the addition of trifluoroacetic acid (final concentration 1%), after which peptides were desalted on a Sep-Pak C18 cartridge (Waters). Samples were dried in a vacuum centrifuge and reconstituted in 2% formic acid for mass spectrometry (MS) analysis. Peptide mixtures were loaded directly on the analytical column (ReproSil-Pur 120 C18-AQ, 1.9 μm, 75 μm × 500 mm, packed in-house) and analyzed by nano liquid chromatography–tandem MS (LC–MS/MS) on an Orbitrap Fusion Tribrid mass spectrometer equipped with a Proxeon nLC1000 system (Thermo Scientific). Solvent A was 0.1% formic acid/water and solvent B was 0.1% formic acid/80% acetonitrile. Peptides were eluted from the analytical column at a constant flow of 250 nl min^–1^ in a 270-min gradient, containing a 250-min stepped increase from 3 to 35% solvent B followed by a 20-min wash in 80% solvent B.

Raw data were analyzed by Proteome Discoverer (v.2.5.0.400, Thermo Scientific) using standard settings. MS/MS data were searched against the Human Swissprot database (20,395 entries, release 2021_04) using Sequest HT. The maximum permitted precursor mass tolerance was 50 ppm and 0.6 Da for fragment ion masses. Trypsin was chosen as cleavage specificity, allowing two missed cleavages. Carbamidomethylation (C) was set as a fixed modification, while oxidation (M) was used as variable modifications. False discovery rates for peptide and protein identification were set to 1% and, as an additional filter, Sequest HT XCorr>1 was set. For wild-type cells, protein peptide spectrum match (PSM) values of PDS5A and PDS5B of three biological replicates were averaged and displayed in the panel.

### Hi-C analysis

Hi-C libraries were prepared as previously described^40^, with the protocol adapted slightly for G1 analyses. An asynchronous pool of cells was first crosslinked using 2% formaldehyde for 10 min at room temperature and quenched with 2 M glycine. The 10% smallest cells were then sorted based on forward and side scatter using a BD FACSAria II. Five million cells were collected for Hi-C analysis and then processed according to a protocol following crosslinking. To assess sorting efficiency, 0.5 million sorted cells and 0.5 million asynchronous cells were permeabilized for 10 min using 0.1% triton in PBS. Cells were stained with DAPI (Sigma-Aldrich) and assayed on a BD LSR Fortessa Machine. Plots were generated with FlowJo (v.10). The gating strategy is depicted in Supplementary Fig. 1a.

Raw sequence data were mapped and processed using HiC-Pro v.2.9 and v.3.0 (ref. ^41^), with hg19 as reference; juicebox-ready files were generated using Juicebox-pre (juicer tools v.1.9.8)^42^ (see Supplementary Table 2 for the number of valid pairs per sample and the percentage of *cis* contacts). For visualization and downstream analyses, contact matrices were ICE normalized^43^ and normalized to 100 million contacts per sample. For Hi-C analysis on asynchronous cells, we first subsampled the data to obtain amounts of reads equal to the sample with the lowest amount of reads.

To visualize the genome-wide effects of our knockout cells, we performed APA^40^ using the loops previously identified in wild-type HAP1 cells^3^. APA was performed as implemented in GENOVA v.1.0 (ref. ^44^). In brief, for the set of loop coordinates a square submatrix was selected centred at these locations, including a 100-kb flanking region upstream and downstream. These submatrices were then averaged to obtain a mean contact map for these locations. The difference plots were obtained as the difference of mean contact maps in comparison with the indicated control cell line. We performed a similar analysis for extended loops, as described previously^3^. Extended loops are defined as those formed when the 5’ loop anchor is combined with every 3’ loop anchor in a 3-Mb region that is not the primary loop itself. APA scores for primary and extended loops were measured using the quantify function with size 3 in GENOVA v.1.0 to obtain the mean signal intensity of each loop.

For analysis of differences in stripes, we used an aggregate analysis similar to those described above, as has been done previously^45^. We selected the top 10,000 CTCF chromatin immunoprecipitation sequencing peaks in wild-type HAP1 cells^11^ as the center location for a submatrix of 6 Mb. To account for differences in distance from the diagonal, the signal was normalized to the expected signal per sample at that distance before averaging them to obtain mean contact maps. In this way we specifically assessed enrichment at stripes, rather than looking at the ‘general’ differences in loop length between genotypes. To quantify differences in stripes across samples, the signal from 3’ and 5’ average stripes was fit to a polynomial surface using local fitting, with stats::loess(), to include the 95% confidence interval in gray.

### Genome-wide synthetic viability screen

*∆HDAC8* HAP1 cells were mutagenized using a gene-trap retrovirus produced in HEK293T cells (obtained from ATCC) and concentrated either by ultracentrifugation as described previously^23^ or employing centrifugal ultrafiltration devices. Here, retrovirus-containing medium was harvested on two consecutive days, filtered (0.45 μm) and concentrated using Amicon Ultra-15 Centrifugal Filter Units with 100 K MWCO (Merck-Millipore). The virus concentrates from both harvests were combined, supplemented with 8 μg ml^–1^ protamine sulfate (Sigma) and used to infect ~40 million HAP1 cells.

To map fitness genes in *ΔHDAC8* HAP1 cells, mutagenized cells were passaged for an additional 14 days after gene-trap virus infection. Cells were then harvested and fixed with BD fix buffer I (BD Biosciences) for 10 min at 37 °C. After washing with FACS buffer (10% FCS in PBS), cells were stained with DAPI (1 μg ml^–1^) for 1 h at room temperature to visualize G1 cells. Twenty-four million G1 haploid cells were sorted using a BD FACSAria Fusion, followed by genomic DNA extraction and library preparation as described in ref. ^23^. The gating strategy is depicted in Supplementary Fig. 1b.

Insertion mapping and data analysis were performed as described previously^23^ with certain modifications. Sequence reads (50 base pairs) were aligned to the human genome (v.hg38) using Bowtie, resulting in a unique alignment to the human genome with zero or one mismatch. Insertions were assigned to protein-coding genes. For every gene, the transcript containing the longest open reading frame was used and unique alignments in intronic regions between the transcription initiation site and stop codon were counted. Genes enriched for gene-trap insertions in either the sense or antisense orientation were identified using a false-discovery-rate-corrected binomial test (step 1, *P* value cutoff 0.05), and genes that deviated in *∆HDAC8* cells from wild-type control cells were identified by a bidirectional Fisher’s exact test with all independent control datasets (step 2, *P* value cutoff 0.05). An odds ratio cutoff of 0.7 was applied using the aggregated wild-type control datasets with a greater Fisher’s test. To find genes whose inactivation rescued the growth defect of *∆HDAC8* cells, we focused on fitness enhancers. These genes are defined by an increase in sense orientation integrations observed in *∆HDAC8* cells but not in wild-type controls (step 1) and show a bias in the sense/antisense ratio compared with all control datasets (step 2). This yielded PDS5A as the strongest and most important fitness enhancer.

### Reporting Summary

Further information on research design is available in the Nature Research Reporting Summary linked to this article.

## Online content

Any methods, additional references, Nature Research reporting summaries, source data, extended data, supplementary information, acknowledgements, peer review information; details of author contributions and competing interests; and statements of data and code availability are available at 10.1038/s41594-022-00773-z.

## Supplementary information

Supplementary Information Supplementary Fig. 1 and Tables 1 and 2.

Reporting Summary

Supplementary Table Gene-trap integration genetic screen. Numbers of gene-trap integrations per genotype in sense and antisense orientation.

## Data Availability

The Hi-C and genetic screen data generated have been deposited in GEO, accession no. GSE174628. The proteomics data have been deposited in the PRIDE database, accession no. PXD032185. Source data are provided with this paper. Any other relevant data are available from the corresponding author upon reasonable request.

## Source data

Source Data Fig. 1 Unprocessed immunoblots.
Source Data Fig. 2 Unprocessed immunoblots.
Source Data Fig. 3 Unprocessed immunoblots.
Source Data Fig. 4 Unprocessed immunoblots.
Source Data Extended Data Fig. 1 Unprocessed immunoblots.
Source Data Extended Data Fig. 3 Unprocessed immunoblots.
Source Data Extended Data Fig. 4 Unprocessed immunoblots.

## Code availability

Hi-C data were analyzed with GENOVA, which can be downloaded here: https://github.com/deWitLab/GENOVA.
