# Supplementary material for: The cohesin acetylation cycle controls chromatin loop length through a PDS5A brake mechanism
Source: Nat Struct Mol Biol. 2022 Jun 16;29(6):586–91. doi: 10.1038/s41594-022-00773-z (PMC9205776; doi:10.1038/s41594-022-00773-z)
Supplement: Supplementary file 1 — Supplementary Fig. 1 and Tables 1 and 2. [file 41594_2022_773_MOESM1_ESM.pdf]

nature portfolio

https://doi.org/10.1038/s41594-022-00773-z

Supplementary information

The cohesin acetylation cycle controls chromatin loop length through a PDS5A brake mechanism

In the format provided by the authors and unedited

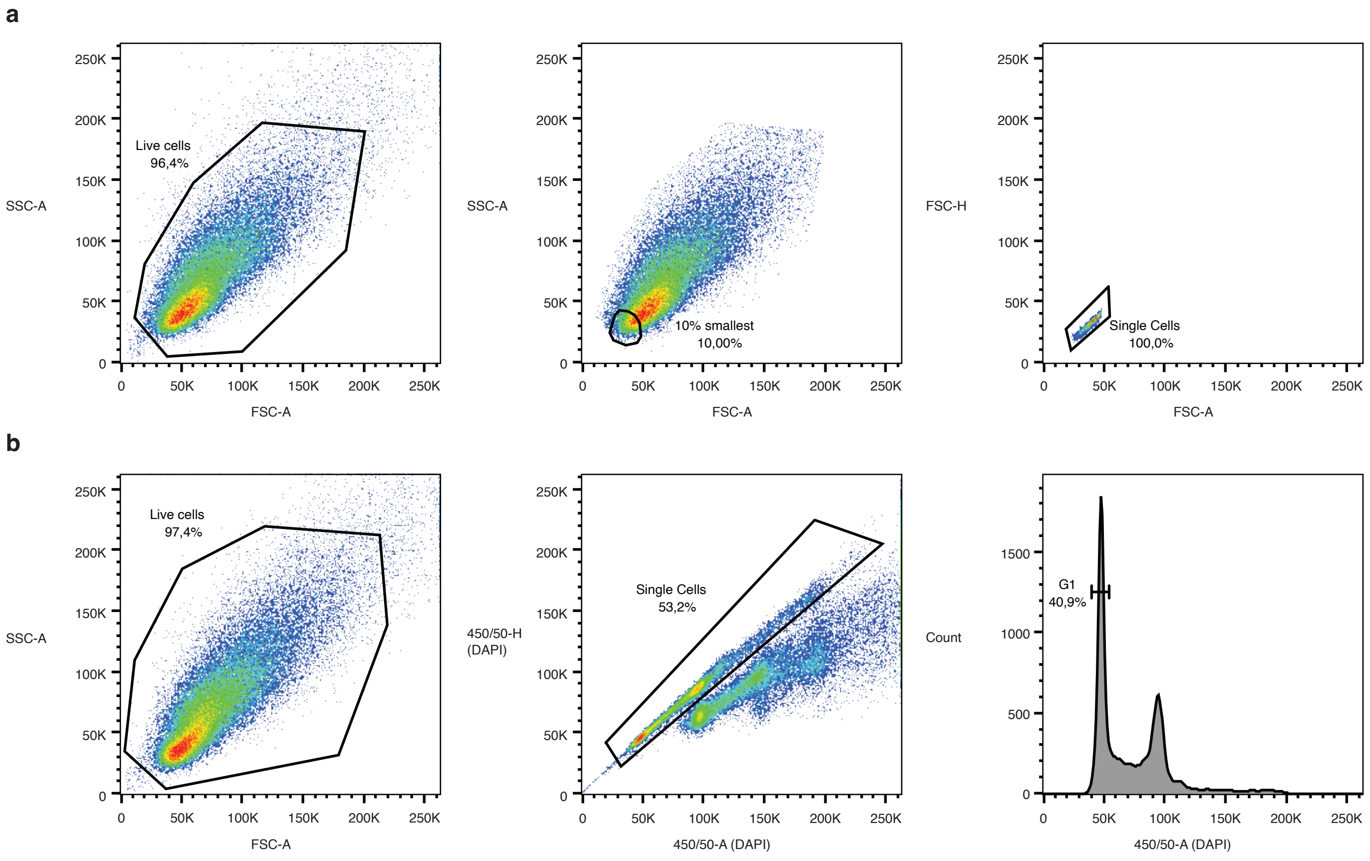

Supplementary Figure 1. FACS-based sorting strategies.
(a) Sorting strategy for Hi-C analysis. Live cells were gated based on FSC/SSC (left). Then the 10% smallest cells were gated based on FSC/SSC (middle). Singlets were gated based on FSC-A/FSC-H (right). Collected cells were then used for Hi-C analysis.
(b) Sorting strategy for the synthetic viability screen. Live cells were gated based on FSC/SSC (left), single cells were gated based on 450/50-A and 450/50-H (DAPI) (middle). Then G1 cells were gated based on the histogram of the DAPI channel (right).

Supplementary Table 1. Description of CRISPRs for generating knockouts.

Cell line	CRISPR	Knockout mechanism
ΔESCO1 clone 1	5'-AGCTTAACCGGAGATCACAA-3'	Blasticidin resistance cassette
ΔESCO1 clone 2	5'-CATGAGTACAAGGTCATCAA-3'	Out of frame deletion
ΔHDAC8 clone 1	5'-CGGGACTATAGATATAAACC-3'	Puromycin resistance cassette
ΔHDAC8 clone 2	5'-CAGTGGGCAGTCGCTGGTCC-3'	Out of frame deletion
ΔESCO1/ΔHDAC8 clone 1	HDAC8: 5'-CGGGACTATAGATATAAACC-3'<br>In *ΔESCO1* clone 1	ESCO1: blasticidin resistance cassette<br>HDAC8: puromycin resistance cassette
ΔESCO1/ΔHDAC8 clone 2	HDAC8: 5'-CAGTGGGCAGTCGCTGGTCC -3'<br>In *ΔESCO1* clone 1	ESCO1: blasticidin resistance cassette<br>HDAC8: puromycin resistance cassette
ΔWAPL/ΔESCO1	ESCO1: 5'-CATGAGTACAAGGTCATCAA-3'<br>In ΔWAPL clone generated previously[3]	WAPL: blasticidin resistance cassette<br>ESCO1: out of frame deletion
ΔPDS5A clone 1	5'-GTGGCGTCGTGAGTGCCGACGGG-3'	Puromycin resistance cassette
ΔPDS5A clone 2	5'-GGAAGATCGCTTACCCTCCG-3'	Puromycin resistance cassette
ΔPDS5A/ΔHDAC8	PDS5A: 5'-GTGGCGTCGTGAGTGCCGACGGG-3'<br>In *ΔHDAC8* clone 1	HDAC8: puromycin resistance cassette<br>PDS5A: blasticidin resistance cassette
ΔPDS5B clone 1	5'-TCTGATATTTCCTTGACCCC-3'	Puromycin resistance cassette
ΔPDS5B clone 2	5'-TCTGATATTTCCTTGACCCC-3'	Puromycin resistance cassette

Gene names are depicted in Italic.

Supplementary Table 2. Hi-C statistics.

Sample	# of read pairs	# valid pairs	% *cis* contacts	% *cis* short (< 20kb)	% cis long (> 20kb)
Wild type G1 replicate 1	100989616	96631266	85.95	15.81	84.19
Wild type G1 replicate 2	198574691	173320705	87.09	18.71	81.29
ΔESCO1 G1 replicate 1	98713428	94292026	84.44	14.57	85.43
ΔESCO1 G1 replicate 2	214202411	180691605	87.17	18.97	81.03
ΔHDAC8 G1 replicate 1	106708953	101695353	83.74	17.86	82.14
ΔHDAC8 G1 replicate 2	179297463	159409238	88.89	19.15	80.85
ΔESCO1/ΔHDAC8 G1 replicate 1	60240911	56367395	86.55	21.87	78.13
ΔESCO1/ΔHDAC8 G1 replicate 2	22425381	21886797	78.74	17.45	82.55
ΔWAPL G1 replicate 1	139857623	133451260	84.59	11.47	88.53
ΔWAPL G1 replicate 2	33384903	32629039	77.73	11.88	88.12
ΔESCO1/ΔWAPL G1 replicate 1	140475681	133306368	89.44	15.47	84.53
ΔESCO1/ΔWAPL G1 replicate 2	33093458	32335960	85.83	14.64	85.36
ΔPDS5A G1 replicate 1	238364672	206191811	84.87	14.77	85.22
ΔPDS5A G1 replicate 2	40673660	39680479	77.59	11.49	88.51
ΔHDAC8/ΔPDS5A G1 replicate 1	232927775	198486088	84.67	15.05	84.95
ΔHDAC8/ΔPDS5A G1 replicate 2	32189252	31447321	81.29	12.81	87.19
ΔPDS5B G1 replicate 1	270313946	218645461	87.09	20.48	79.52
ΔPDS5B G1 replicate 2	18432949	18024477	79.11	17.76	82.24

Wild type G1 replicate 3	46287992	44802525	81.80	12.62	87.38
Wild type G1 replicate 4	40391970	39412588	73.41	12.33	87.67
Wild type G1 replicate 5	19481766	19029023	78.94	16.48	83.52
Wild Type Asynchronous	58929363	47376183	83.92	16.19	83.81
ΔHDAC8 Asynchronous	315335182	245254374	84.33	15.44	84.56

Gene names are depicted in Italic.
