# Supplementary material for: The cohesin acetylation cycle controls chromatin loop length through a PDS5A brake mechanism
Source: Nat Struct Mol Biol. 2022 Jun 16;29(6):586–91. doi: 10.1038/s41594-022-00773-z (PMC9205776; doi:10.1038/s41594-022-00773-z)
Supplement: Supplementary file 2 — Reporting Summary [file 41594_2022_773_MOESM2_ESM.pdf]

nature research

Corresponding author(s): Benjamin Rowland

Last updated by author(s): 03/14/2022

# Reporting Summary

Nature Research wishes to improve the reproducibility of the work that we publish. This form provides structure for consistency and transparency in reporting. For further information on Nature Research policies, see our Editorial Policies and the Editorial Policy Checklist.

## Statistics

For all statistical analyses, confirm that the following items are present in the figure legend, table legend, main text, or Methods section.

| n/a | Confirmed | |
|---|---|---|
| ☐ | ☒ | The exact sample size (*n*) for each experimental group/condition, given as a discrete number and unit of measurement |
| ☐ | ☒ | A statement on whether measurements were taken from distinct samples or whether the same sample was measured repeatedly |
| ☐ | ☒ | The statistical test(s) used AND whether they are one- or two-sided<br>*Only common tests should be described solely by name; describe more complex techniques in the Methods section.* |
| ☒ | ☐ | A description of all covariates tested |
| ☒ | ☐ | A description of any assumptions or corrections, such as tests of normality and adjustment for multiple comparisons |
| ☐ | ☒ | A full description of the statistical parameters including central tendency (e.g. means) or other basic estimates (e.g. regression coefficient) AND variation (e.g. standard deviation) or associated estimates of uncertainty (e.g. confidence intervals) |
| ☐ | ☒ | For null hypothesis testing, the test statistic (e.g. *F*, *t*, *r*) with confidence intervals, effect sizes, degrees of freedom and *P* value noted<br>*Give P values as exact values whenever suitable.* |
| ☒ | ☐ | For Bayesian analysis, information on the choice of priors and Markov chain Monte Carlo settings |
| ☒ | ☐ | For hierarchical and complex designs, identification of the appropriate level for tests and full reporting of outcomes |
| ☒ | ☐ | Estimates of effect sizes (e.g. Cohen's *d*, Pearson's *r*), indicating how they were calculated |

*Our web collection on statistics for biologists contains articles on many of the points above.*

## Software and code

Policy information about availability of computer code

| | |
|---|---|
| Data collection | Hi-C sequencing data was processed with HiC-Pro 2.9 and 3.0. HiC-Pro output was converted to juicer files using juicebox-pre (juicer tools v1.9.8).<br>Images for FRAP analysis were acquired using the Leica SP5 Confocal microscope 63x/1.32 oil lens using LAS-AF Software (Leica).<br>For mass spectometry analysis, peptides were analyzed by nanoLC-MS/MS on an Orbitrap Fusion Tribrid mass spectrometer equipped with a Proxeon nLC1000 system (Thermo Scientific). |
| Data analysis | Hi-C data analysis was performed with GENOVA v1.0 (van der Weide et al., 2021, NAR Genom Bioinform).<br>For FRAP analysis, the fluorescence intensity was measured in the bleached and unbleached area by user-defined regions in ImageJ V2.1.0/1.53k.<br>For mass spectometry analysis, the raw data was analyzed by Proteome Discoverer (PD) (version 2.5.0.400, Thermo Scientific) using standard settings. MS/MS data were searched against the Human Swissprot database (20,395 entries, release 2021_04) using Sequest HT. |

For manuscripts utilizing custom algorithms or software that are central to the research but not yet described in published literature, software must be made available to editors and reviewers. We strongly encourage code deposition in a community repository (e.g. GitHub). See the Nature Research guidelines for submitting code & software for further information.

## Data

Policy information about availability of data

All manuscripts must include a data availability statement. This statement should provide the following information, where applicable:

- Accession codes, unique identifiers, or web links for publicly available datasets
- A list of figures that have associated raw data
- A description of any restrictions on data availability

The generated Hi-C data has deposited in GEO (accession number GSE174628).
The generated mass spectometry data has been depositied in PRIDE (accession number PXD032185).

# Field-specific reporting

Please select the one below that is the best fit for your research. If you are not sure, read the appropriate sections before making your selection.

☒ Life sciences ☐ Behavioural & social sciences ☐ Ecological, evolutionary & environmental sciences

For a reference copy of the document with all sections, see nature.com/documents/nr-reporting-summary-flat.pdf

# Life sciences study design

All studies must disclose on these points even when the disclosure is negative.

| | |
|---|---|
| Sample size | G1 Hi-C library preparation was performed in duplicate for all genotypes: Wild Type, ΔESCO1, ΔHDAC8, ΔESCO1/ΔHDAC8, ΔWAPL, ΔWAPL/ΔESCO1, ΔPDS5A, ΔPDS5A/ΔHDAC8, and ΔPDS5B cells. Two independent clones were used for ΔESCO1, ΔHDAC8, ΔESCO1/ΔHDAC8, ΔPDS5A, and ΔPDS5B cells. The same clone was used for ΔWAPL, ΔWAPL/ΔESCO1, and ΔPDS5A/ΔHDAC8 cells for an independent Hi-C experiment. Hi-C analysis was performed once on asynchronous Wild Type and ΔHDAC8 cells. No sample size calculations were performed. Sample sizes were chosen based on common standards of the field. |
| Data exclusions | No data was excluded in our analysis. |
| Replication | G1 Hi-C library preparation was performed in duplicate for all described genotypes. Replicates yielded similar phenotypes. Hi-C library preparation was performed once for asynchronous Wild Type and ΔHDAC8 cells. The asynchronous Hi-C analysis was only performed once as we used these results as a starting point for this study. We validated this role for HDAC8 in 3D genome organization specifically by performing Hi-C analysis in G1 cells. We did perform replicates for all the G1 Hi-C experiments.<br>Co-immunoprecipitation experiments were performed in triplicate. All attempts at replication were succesfull.<br>Haploid genetic screen in ΔHDAC8 cells has been performed once in this study, and is compared to 4 independent Wild Type screens that were published earlier (see methods). The screen in ΔHDAC8 cells was performed once, as we only used this screen to identify potential new regulators. To study these genetic interactions, we generated knockout cell lines and performed subsequent Hi-C analysis/Co-IP experiments. We performed replicates for each of these follow-up experiments. |
| Randomization | Randomization is not relevant to this study, as samples were not subdivided into different experimental groups. |
| Blinding | Blinding is not relevant to this study, as samples were not subdivided into different experimental groups. |

# Reporting for specific materials, systems and methods

We require information from authors about some types of materials, experimental systems and methods used in many studies. Here, indicate whether each material, system or method listed is relevant to your study. If you are not sure if a list item applies to your research, read the appropriate section before selecting a response.

### Materials & experimental systems

| n/a | Involved in the study |
|---|---|
| ☐ | ☒ Antibodies |
| ☐ | ☒ Eukaryotic cell lines |
| ☒ | ☐ Palaeontology and archaeology |
| ☒ | ☐ Animals and other organisms |
| ☒ | ☐ Human research participants |
| ☒ | ☐ Clinical data |
| ☒ | ☐ Dual use research of concern |

### Methods

| n/a | Involved in the study |
|---|---|
| ☒ | ☐ ChIP-seq |
| ☐ | ☒ Flow cytometry |
| ☒ | ☐ MRI-based neuroimaging |

## Antibodies

| | |
|---|---|
| Antibodies used | Co-immunoprecipitation experiments were performed with the following antibodies: SMC1 (Bethyl, A300-055A) and SCC2 (Bethyl, |

| | |
|---|---|
| Antibodies used | A301-779A). Western Blots were performed using the following antibodies and dilutions: HSP90 (Santa Cruz, sc13119 F8, 1:10.000), ESCO1 (a kind gift from Susanna Rankin (Oklahoma Medical Research Foundation), 1:1500), HDAC8 (Sigma-Aldrich, WH0055869M1, 1:1000), AcSMC3 (a kind gift from Katsuhiko Shirahige (The University of Tokyo), 1:1500), WAPL (Santacruz, sc365189, 1:1000), SMC1 (Bethyl, A300-055A, 1:2000), SMC3 (Bethyl, A300-060A-5, 1:2000), SCC1 (Millipore, 05-908, 1:1000), PDS5A (Bethyl, A300-089A, 1:1000), PDS5B (Bethyl, A300-538A, 1:500), SCC2 (Santa Cruz, sc374625, 1:1000), SCC4 (Abcam, ab46906, 1:1000), Actin (Abcam, ab6276, 1:5000), and Tubulin (Abcam, ab18251, 1:10.000). Secondary antibodies Goat-anti-Mouse-PO (DAKO, P0447) and Goat-anti-Rabbit-PO (DAKO, P0448) were used at 1:2000 dilution. |
| Validation | Validation information can be found at the following websites for the following proteins:<br>HSP90: https://www.scbt.com/p/hsp-90alpha-beta-antibody-f-8<br>HDAC8: https://www.sigmaaldrich.com/catalog/product/sigma/wh0055869m1?lang=en&region=NL<br>SMC1: https://www.bethyl.com/product/A300-055A/SMC1+Antibody<br>SMC3: https://www.bethyl.com/product/A300-060A/SMC3+Antibody<br>SCC1: https://www.merckmillipore.com/NL/en/product/Anti-RAD21-Antibody,MM_NF-05-908<br>PDS5A: https://www.bethyl.com/product/A300-089A/SCC-112+Antibody<br>Tubulin: https://www.abcam.com/alpha-tubulin-antibody-microtubule-marker-ab18251.html<br>Goat-anti-Mouse: https://www.agilent.com/store/en_US/Prod-P044701-2/P044701-2<br>Goat-anti-Rabbit: https://www.agilent.com/store/en_US/Prod-P044801-2/P044801-2<br>WAPL: https://www.scbt.com/p/wapl-antibody-a-7<br>SCC2 (IP): https://www.bethyl.com/product/A301-779A/NIPBL+Antibody<br>SCC2 (WB): https://www.scbt.com/p/nipbl-antibody-c-9<br>SCC4: https://www.abcam.com/scc4-antibody-ab46906.html<br>PDS5B: https://www.bethyl.com/product/A300-538A/Pds5B+Antibody<br>Actin: https://www.abcam.com/beta-actin-antibody-ac-15-ab6276.html<br>The following two antibodies were kind gifts from Susanna Ranking (Oklahoma Medical Research Foundation) and Katsuhuko Shirahige (The University of Tokyo). They are validated in the papers below.<br>ESCO1: Alomer, R. M. et al., Proc Natl Acad Sci USA 114, 9906–9911 (2017).<br>AcSMC3: Nishiyama, T. et al., Cell 143, 737–749 (2010). |

## Eukaryotic cell lines

Policy information about cell lines

| | |
|---|---|
| Cell line source(s) | HAP1 Wild Type cells from Carette et al., Nature 2011, a gift from the authors.<br>ΔWAPL HAP1 cells from Haarhuis et al., 2017 Cell, a gift from the authors.<br>All other HAP1 knockouts cells were generated in this study using CRISPR/Cas9 gene editing.<br>HEK293T cells were obtained from ATCC. |
| Authentication | The presence of a resistance casette or indels was confirmed by PCR and Sanger sequencing. The absence of protein was confirmed by Western Blotting Analysis. |
| Mycoplasma contamination | All cell lines were negative for mycoplasma contamination. |
| Commonly misidentified lines<br>(See ICLAC register) | No commonly misidentified cell line was used. |

## Flow Cytometry

### Plots

Confirm that:

☒ The axis labels state the marker and fluorochrome used (e.g. CD4-FITC).

☒ The axis scales are clearly visible. Include numbers along axes only for bottom left plot of group (a 'group' is an analysis of identical markers).

☒ All plots are contour plots with outliers or pseudocolor plots.

☒ A numerical value for number of cells or percentage (with statistics) is provided.

### Methodology

| | |
|---|---|
| Sample preparation | Hi-C Analysis: Hi-C libraries were prepared as previously described (Rao et al., 2014 Cell). The protocol was adapted slightly for G1 analyses. An asynchronous pool of cells was first crosslinked using 2% formaldehyde. Then the 10% smallest cells were sorted based on Forward Scatter and Side Scatter using a BD FACSAria II. 5 million cells were collected for Hi-C analysis and then processed according to protocol following crosslinking. To assess the sorting efficiency, 0.5 million sorted cells and 0.5 million asynchronous cells were permeabilized for 10 minutes using 0.1% triton in PBS. Cells were stained with DAPI (Sigma-Aldrich) and assayed on the BD LSR Fortessa Machine. Plots were generated with FlowJo (v10).<br>Screen: ΔHDAC8 cells were harvested and fixed with BD fix buffer I (BD Biosciences) for 10 minutes at 37C. After washing with FACS buffer (10% FCS in PBS), cells were stained with DAPI (1 ug/ml) for 1 hour at room temperature to visualize G1 cells. 24 million G1 haploid cells were sorted using a BD FACSAria Fusion, followed by gDNA extraction and library preparation as described in (Blomen et al., 2015 Science). |
| Instrument | Sorting for Hi-C Analysis: BD FACSAria II |

| | |
|---|---|
| Instrument | Sorting for screen: BD FACSAria Fusion |
| Software | FlowJo (v10) |
| Cell population abundance | Hi-C Analysis: Each post-sort fraction was the smallest 10% of the single cells from the sample. Extra cells have been sorted and stained with DAPI to check the purity of the sample.<br>Screen: The post sort fraction were the cells that fell into the G1 gate based on the histogram for the DAPI channel. |
| Gating strategy | Hi-C Analysis: Live cells were gated based on FSC/SSC, 10% smallest cells were gated based on 100% live cells in FSC/SSC plot. Then, singlets were gated based on FSC-A/FSC-H.<br>Screen: Live cells were gated based on FSC/SSC, single cells were gated based on 450/50-A/450/50-H (DAPI), G1 cells were gated based on DAPI histogram. |

☒ Tick this box to confirm that a figure exemplifying the gating strategy is provided in the Supplementary Information.
