# Supplementary material for: The cohesin acetylation cycle controls chromatin loop length through a PDS5A brake mechanism
Source: Nat Struct Mol Biol. 2022 Jun 16;29(6):586–91. doi: 10.1038/s41594-022-00773-z (PMC9205776; doi:10.1038/s41594-022-00773-z)
Supplement: Source Data Fig. 1 — Unprocessed immunoblots. [file 41594_2022_773_MOESM4_ESM.pdf]

Figure 1a

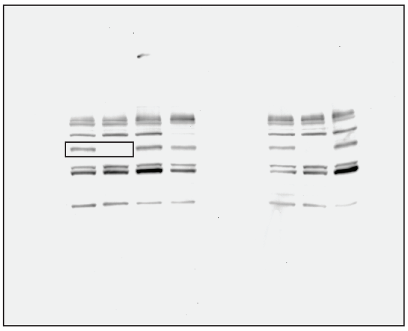

Rabbit-anti-ESCO1

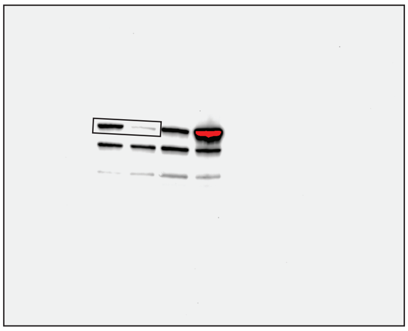

Mouse-anti-AcSMC3

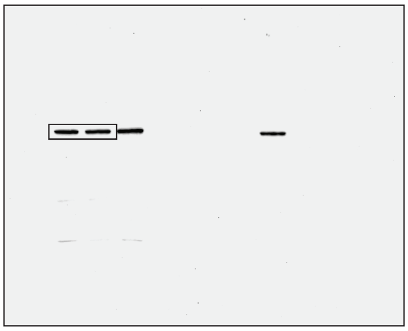

Rabbit-anti-SMC3

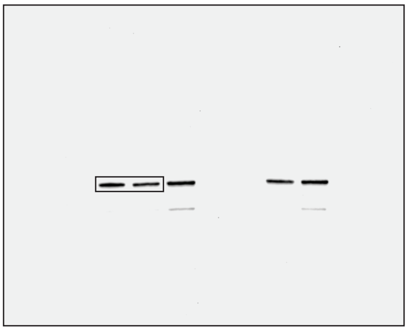

Mouse-anti-SCC1

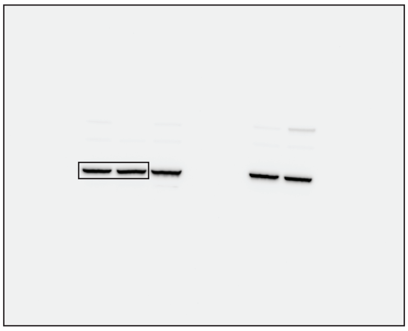

Mouse-anti-HSP90

Figure 1b

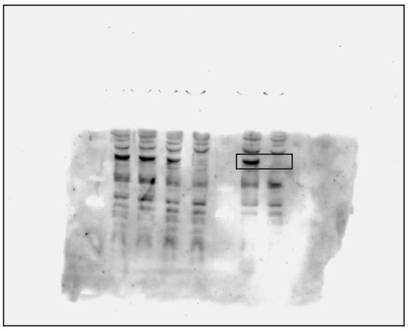

Mouse-anti-HDAC8

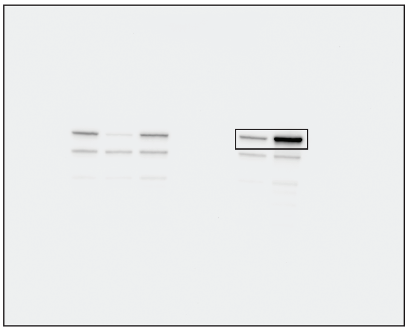

Mouse-anti-AcSMC3

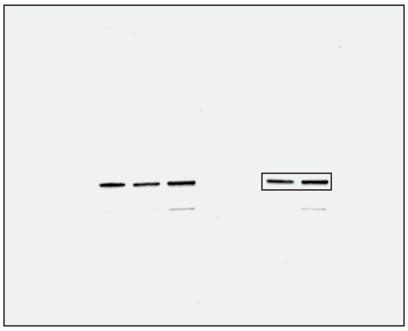

Mouse-anti-SCC1

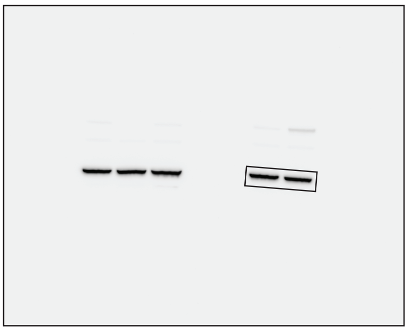

Mouse-anti-HSP90
