# Supplementary figures and images for: The cohesin acetylation cycle controls chromatin loop length through a PDS5A brake mechanism
Source: Nat Struct Mol Biol. 2022 Jun 16;29(6):586–91. doi: 10.1038/s41594-022-00773-z (PMC9205776; doi:10.1038/s41594-022-00773-z)

**Figure 2b**

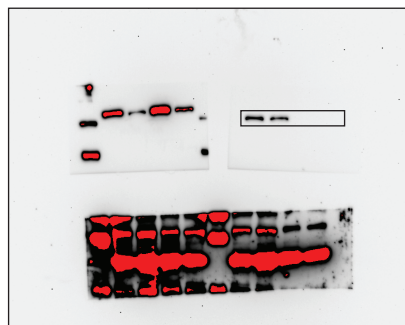

Mouse-anti-WAPL

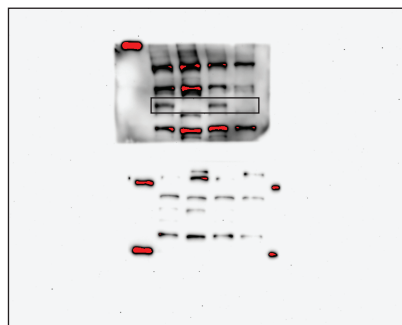

Rabbit-anti-ESCO1

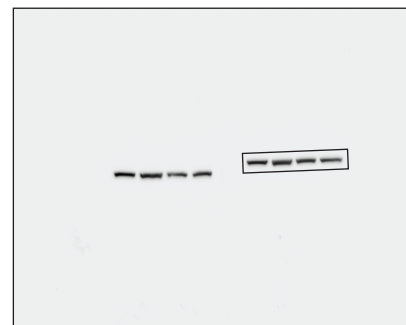

Mouse-anti-HSP90

Supplement: Source Data Fig. 2 — Unprocessed immunoblots. [file 41594_2022_773_MOESM5_ESM.pdf]

**Figure 3c**

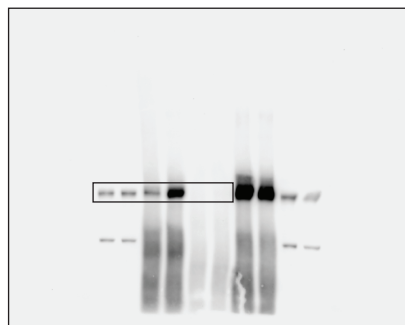

Rabbit-anti-PDS5A

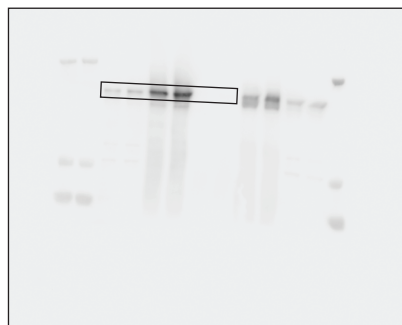

Rabbit-anti-SMC1

Supplement: Source Data Fig. 3 — Unprocessed immunoblots. [file 41594_2022_773_MOESM6_ESM.pdf]

**Figure 4a**

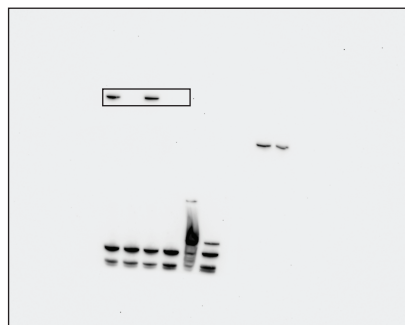

Rabbit-anti-PDS5A

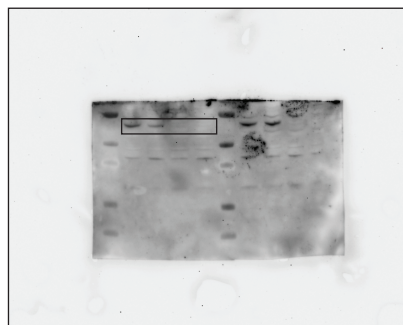

Mouse-anti-HDAC8

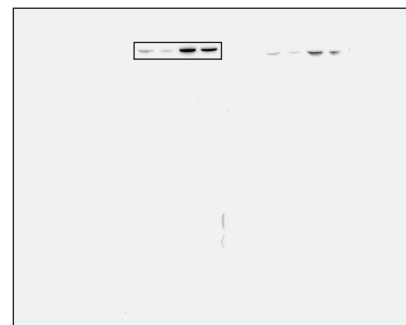

Mouse-anti-AcSMC3

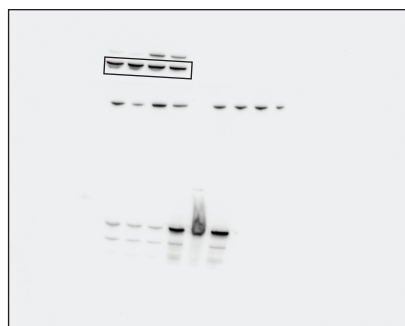

Mouse-anti-HSP90

Supplement: Source Data Fig. 4 — Unprocessed immunoblots. [file 41594_2022_773_MOESM7_ESM.pdf]

# Extended Data Figure 1d

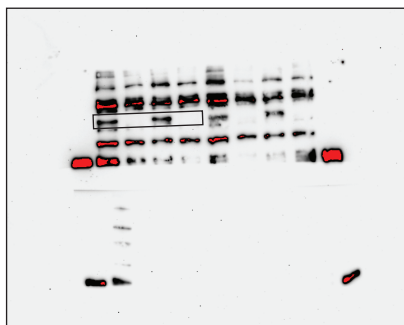

Rabbit-anti-ESCO1

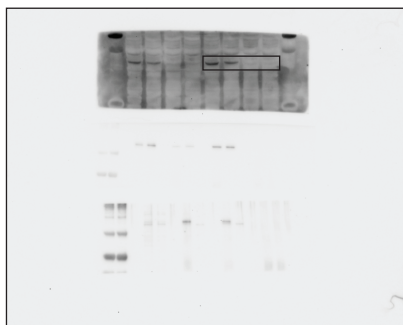

Mouse-anti-HDAC8

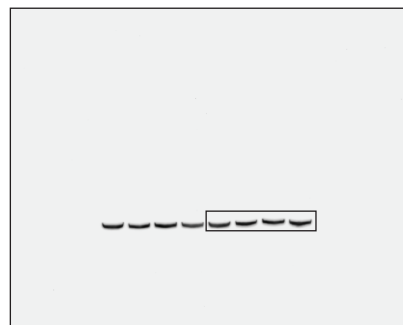

Mouse-anti-HSP90

Supplement: Source Data Extended Data Fig. 1 — Unprocessed immunoblots. [file 41594_2022_773_MOESM8_ESM.pdf]
