# Supplementary material for: The cohesin acetylation cycle controls chromatin loop length through a PDS5A brake mechanism
Source: Nat Struct Mol Biol. 2022 Jun 16;29(6):586–91. doi: 10.1038/s41594-022-00773-z (PMC9205776; doi:10.1038/s41594-022-00773-z)
Supplement: Source Data Extended Data Fig. 3 — Unprocessed immunoblots. [file 41594_2022_773_MOESM9_ESM.pdf]

Extended Data Figure 3d

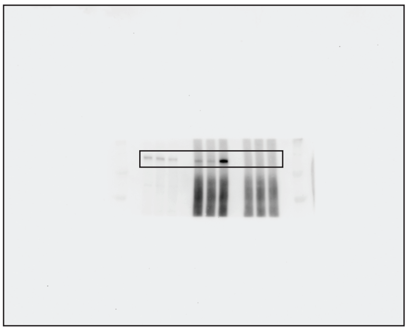

Rabbit-anti-PDS5A

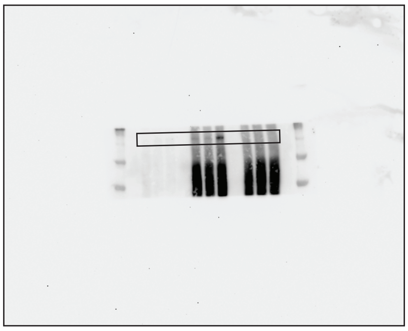

Rabbit-anti-PDS5B

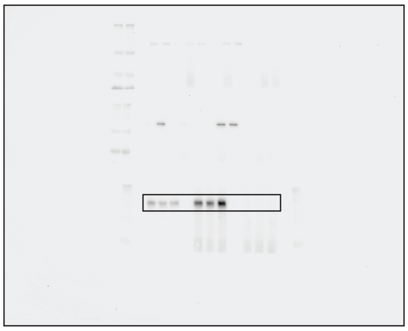

Mouse-anti-WAPL

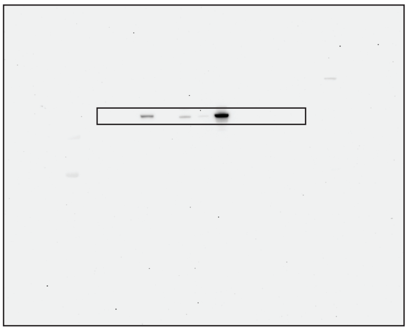

Mouse-anti-AcSMC3

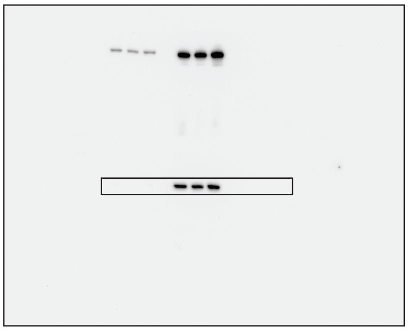

Rabbit-anti-SMC3

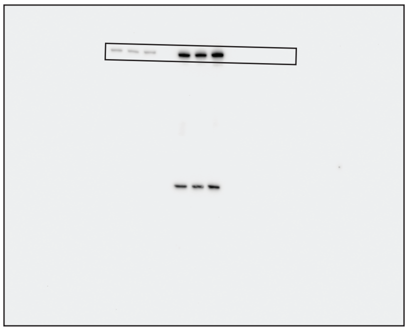

Rabbit-anti-SMC1

Extended Data Figure 3e

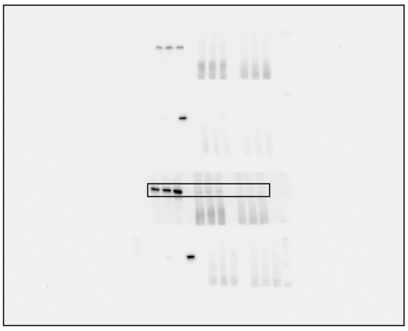

Rabbit-anti-SMC1 (short exposure)

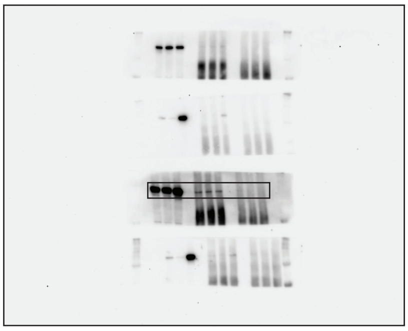

Rabbit-anti-SMC1 (long exposure)

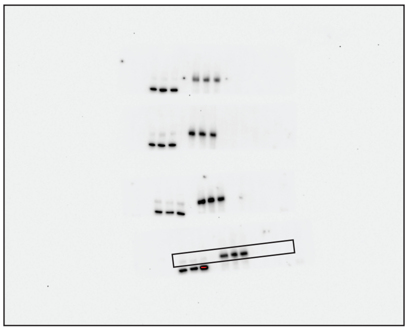

Mouse-anti-SCC2^{NIPBL}

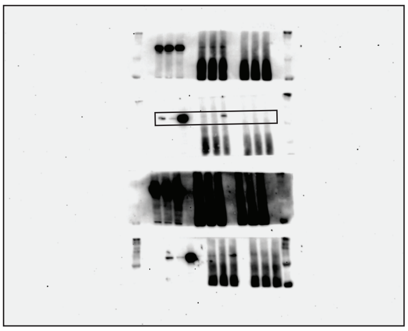

Mouse-anti-AcSMC3

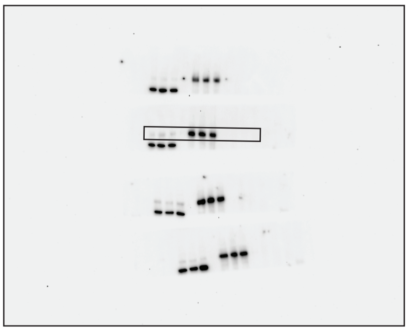

Mouse-anti-SCC2^{NIPBL}
