# Supplementary material for: The cohesin acetylation cycle controls chromatin loop length through a PDS5A brake mechanism
Source: Nat Struct Mol Biol. 2022 Jun 16;29(6):586–91. doi: 10.1038/s41594-022-00773-z (PMC9205776; doi:10.1038/s41594-022-00773-z)
Supplement: Source Data Extended Data Fig. 4 — Unprocessed immunoblots. [file 41594_2022_773_MOESM10_ESM.pdf]

Extended Data Figure 4g

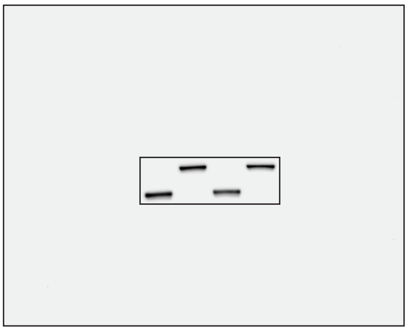
Mouse-anti-SCC1

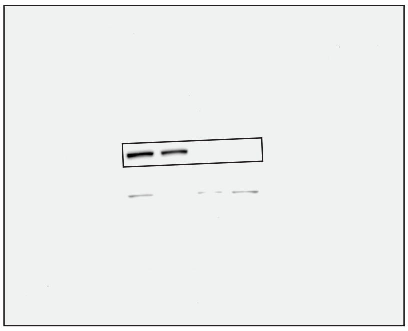
Rabbit-anti-PDS5A

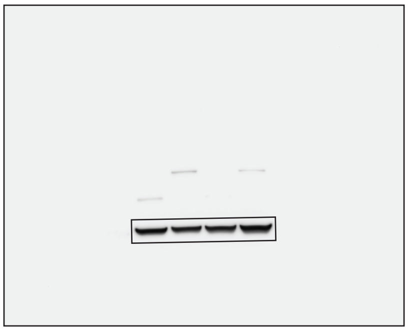
Mouse-anti-HSP90

Extended Data Figure 4j

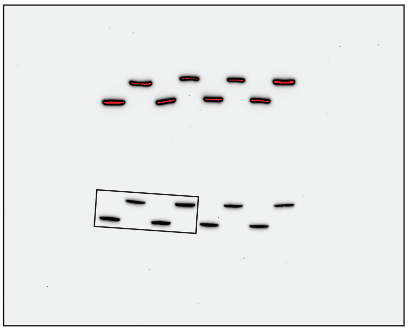
Mouse-anti-SCC1

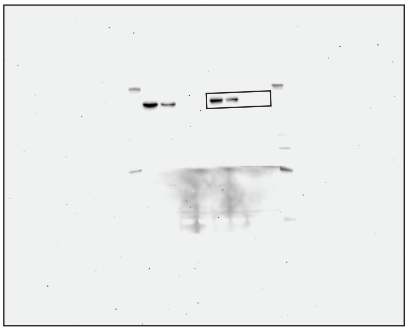
Rabbit-anti-PDS5B

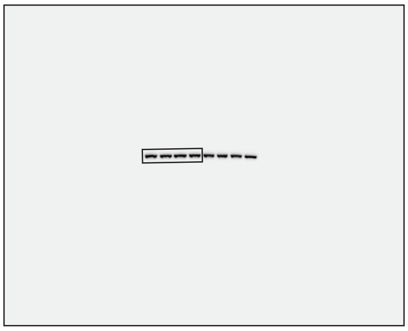
Mouse-anti-Actin
